# Effect of extradural constriction on CSF flow in rat spinal cord

**DOI:** 10.1186/s12987-019-0127-8

**Published:** 2019-03-26

**Authors:** Joel A. Berliner, Thomas Woodcock, Elmira Najafi, Sarah J. Hemley, Magdalena Lam, Shaokoon Cheng, Lynne E. Bilston, Marcus A. Stoodley

**Affiliations:** 1 Faculty of Medicine and Health Sciences, Macquarie University, Technology Place, Sydney, NSW 2109 Australia; 2 Elsevier Inc, John F Kennedy Boulevard, Philadelphia, PA 19103 USA; 3 Department of Engineering, Faculty of Science and Engineering, Macquarie University, Sydney, NSW 2109 Australia; 4 Neuroscience Research Australia, Margarete Ainsworth Building, Barker Street, Sydney, NSW 2031 Australia; 5 Prince of Wales Clinical School, University of New South Wales, Sydney, NSW 2031 Australia

**Keywords:** Cerebrospinal fluid, Spinal cord, Perivascular, Syringomyelia

## Abstract

**Background:**

Fluid homeostasis in the central nervous system (CNS) is essential for normal neurological function. Cerebrospinal fluid (CSF) in the subarachnoid space and interstitial fluid circulation in the CNS parenchyma clears metabolites and neurotransmitters and removes pathogens and excess proteins. A thorough understanding of the normal physiology is required in order to understand CNS fluid disorders, including post-traumatic syringomyelia. The aim of this project was to compare fluid transport, using quantitative imaging of tracers, in the spinal cord from animals with normal and obstructed spinal subarachnoid spaces.

**Methods:**

A modified extradural constriction model was used to obstruct CSF flow in the subarachnoid space at the cervicothoracic junction (C7–T1) in Sprague–Dawley rats. Alexa-Fluor 647 Ovalbumin conjugate was injected into the cisterna magna at either 1 or 6 weeks post–surgery. Macroscopic and microscopic fluorescent imaging were performed in animals sacrificed at 10 or 20 min post–injection. Tracer fluorescence intensity was compared at cervical and thoracic spinal cord levels between control and constriction animals at each post-surgery and post-injection time point. The distribution of tracer around arterioles, venules and capillaries was also compared.

**Results:**

Macroscopically, the fluorescence intensity of CSF tracer was significantly greater in spinal cords from animals with a constricted subarachnoid space compared to controls, except at 1 week post-surgery and 10 min post-injection. CSF tracer fluorescence intensity from microscopic images was significantly higher in the white matter of constriction animals 1 week post surgery and 10 min post-injection. At 6 weeks post–constriction surgery, fluorescence intensity in both gray and white matter was significantly increased in animals sacrificed 10 min post-injection. At 20 min post-injection this difference was significant only in the white matter and was less prominent. CSF tracer was found predominantly in the perivascular spaces of arterioles and venules, as well as the basement membrane of capillaries, highlighting the importance of perivascular pathways in the transport of fluid and solutes in the spinal cord.

**Conclusions:**

The presence of a subarachnoid space obstruction may lead to an increase in fluid flow within the spinal cord tissue, presenting as increased flow in the perivascular spaces of arterioles and venules, and the basement membranes of capillaries. Increased fluid retention in the spinal cord in the presence of an obstructed subarachnoid space may be a critical step in the development of post-traumatic syringomyelia.

**Electronic supplementary material:**

The online version of this article (10.1186/s12987-019-0127-8) contains supplementary material, which is available to authorized users.

## Background

Cerebrospinal fluid (CSF) is a clear, colorless fluid that bathes the central nervous system (CNS). It has several critical functions, including maintenance of a homeostatic environment for neurons and glia, transport of neuroactive substances around the CNS, and acting as a drainage system for CNS interstitial fluid [1–6]. The classical view of CSF circulation is that it is produced primarily in the ventricles by the choroid plexus, and flows into the subarachnoid space surrounding the brain and spinal cord, finally being reabsorbed through arachnoid granulations in the superior sagittal sinus, across the cribriform plate, or via spinal arachnoid villi [3, 7, 8]. Efflux of subarachnoid CSF may also occur via cranial and spinal nerves [9]. In addition to this ‘macrocirculation’, there is an interchange of CSF and interstitial fluid (ISF), allowing for a ‘microcirculation’ of ISF through the CNS parenchyma. This route of fluid flow potentially brings neuroactive substances into contact with neuronal cells and facilitates the removal of waste products. The details of this interchange are still poorly understood [5, 6, 10].

Recent studies suggest that CSF enters the brain parenchyma along periarterial spaces and exits around perivenular spaces, and that this pathway could be important for the clearance of metabolites from the parenchyma [11, 12]. However, compelling evidence of a distinct bulk flow pathway for CSF/ISF exchange has not been produced. Other studies have indicated different drainage pathways, where interstitial fluid and solutes of the brain flow outwards via capillary basal laminae and smooth muscle basement membranes of arterioles and arteries [13, 14]. This route has been described as ‘intramural periarterial drainage’ [15]. Still, even less is known about the route of CSF flow and exchange in the spinal cord. In ovine and rodent models, spinal subarachnoid CSF flows rapidly into large perivascular spaces that funnel into small perivascular spaces of the central gray matter toward the central canal. In the adjacent ECS, limited mixing of fluid tracers with ISF occurs [16, 17]. In rodent models of syringomyelia, there is also rapid flow from the spinal subarachnoid space into perivascular spaces [18, 19]. Computational models suggest that arterial pulsations are a key factor in fluid flow in the perivascular spaces [20, 21], raising the possibility that alterations in pulse wave timing or amplitude could increase fluid inflow.

Syringomyelia is a condition in which high-pressure fluid-filled cysts (syrinxes) form in the spinal cord leading to weakness, pain, and paralysis [22, 23]. Little is known regarding the mechanisms of formation and enlargement of a syrinx, or indeed the source of the fluid. Post–traumatic syringomyelia is typically associated with a narrowing or obstruction in the subarachnoid space after spinal cord injury, but how this leads to formation of a fluid-filled cavity is unknown. Current surgical treatment is not always effective, and syrinx recurrence rate can be as high as 50% [24–30]. There is therefore a need to improve our understanding of fluid inflow and outflow pathways in the spinal cord. In this study, we tested the hypothesis that a subarachnoid obstruction changes the pattern of fluid flow into and through the spinal cord by increasing the inflow at the level of obstruction. This was investigated using a fluorescent tracer to examine fluid flow in the spinal cord of normal animals and in the presence of an extradural constriction.

## Materials and methods

This study was approved by the Animal Care and Ethics Committee of Macquarie University (ARA2013/047). A total of 47 male Sprague–Dawley rats weighing 300–400 g were divided into two groups: experimental animals and control animals. Of these, 28 animals underwent extradural constriction surgery to obstruct the subarachnoid space at the C7–T1 spinal cord level and 19 control animals underwent laminectomy only. At either 1 or 6 weeks post–surgery, the CSF tracer, Alexa–Fluor^®^ 647 ovalbumin, was injected into the CSF circulation at the cisterna magna for 10 or 20 min before sacrifice. Macroscopic and microscopic imaging procedures were carried out on these animals. Post-surgery time points were chosen based on the expected structural changes to subarachnoid space and parenchyma with an acute (1 week) and a chronic (6 weeks) obstruction. The maximum post-surgery time was chosen as 6 weeks to avoid cyst development, as cyst development was previously found between 8 and 13 weeks after extradural constriction [31]. Sacrifice time points of 10 or 20 min post-injection were chosen to observe CSF tracer inflow with minimal outflow. The obstruction was expected to alter inflow of CSF, so time points longer than 20 min would likely demonstrate a combination of inflow and outflow.

### Extradural constriction surgery

**Fig. 1:**
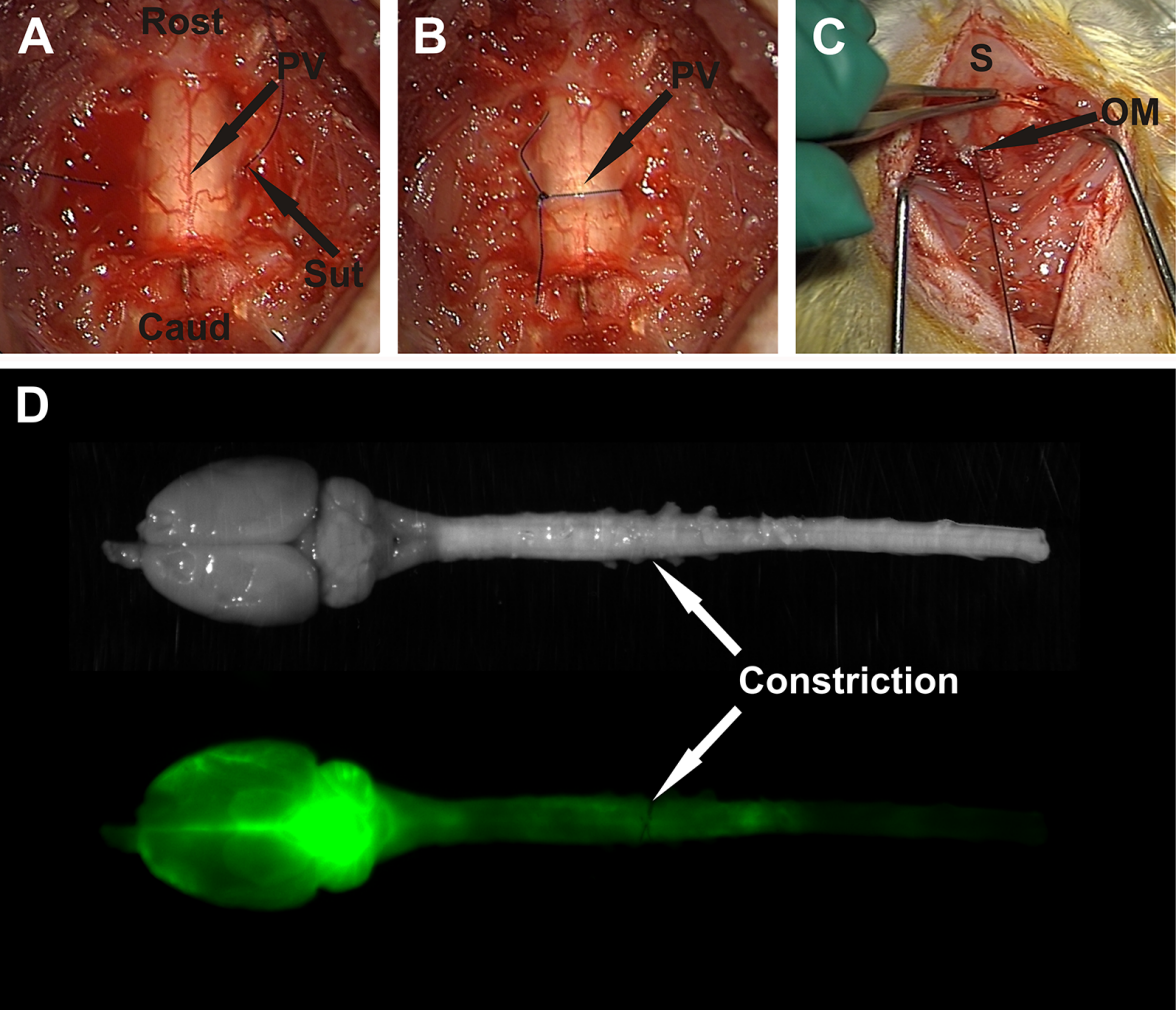
Surgical procedures, and white light and fluorescent imaging of ex vivo brain and spinal cord. The extradural constriction surgeries involved passing a 6-0 monofilament suture (Sut) around the spinal cord (**A**) and tightening the suture to visibly occlude blood flow in the posterior vein (PV; **B**). Exposure of the occipital membrane (OM) for injection of fluorescent tracer into the cisterna magna (**C**). Images of the whole spinal cord and brain under white-light and fluorescent light, with extradural constriction evident (**D**). Labelling: Rost: rostral; Caud: caudal; S: skull

All procedures were performed in a sterile field under general anesthesia induced with 5% isoflurane in oxygen (1L/min) and maintained with 2–2.5% isoflurane through a nose cone. Animals were placed prone, and the skin was shaved and prepared with povidone iodine. An incision was made over the cervicothoracic junction and C7–T1 laminectomies were performed. A 6-0 monofilament suture was passed around the spinal cord outside the dura, tightened until blood flow in the posterior vein was occluded, and then tied with a reef knot (Fig. 1A, B). The wound was closed with 4-0 Absorbable Coated Vicryl sutures (Ethicon, Johnson & Johnson Medical Pacific Pty Ltd, Sydney, Australia). After the operation 0.05 mg/kg of 300 µg/mL buprenorphine in 5% glucose solution was administered subcutaneously. Subsequent doses were given as needed. This surgery was modified from the spinal thecal sac constriction model created by Josephson and colleagues [31]. In the original model the constriction surgery was performed at T8 and the subarachnoid space was obstructed with a 3-0 silk suture.

The animals were allowed food and water ad libitum and monitored for any signs of excessive weight loss, limb weakness, urinary retention or excessive self-grooming.

### CSF tracer injection

At the designated time point, 1 or 6 weeks post-surgery, animals were re-anesthetized. A midline incision was made in the cranio–cervical area to expose the atlanto-occipital membrane. The neck of the animal was flexed to ensure that the atlanto-occipital membrane was taut. Using a stereotactic micromanipulator frame, a 10 µL microsyringe with a bevelled 30G needle (SGE International Pty Ltd, VIC, Australia) was inserted into the cisterna magna and withdrawn slightly to tent-up the atlanto–occipital membrane. A 5 µL injection of the CSF tracer, ovalbumin Alexa–Fluor 647 conjugate (OA-647; Molecular weight 45 kDa; Life Technologies, Victoria, Australia) at a concentration of 20 µg/µL was slowly administered into the cisterna magna. The injection was performed over 1 min and the needle was then left in-place to prevent CSF leakage (Fig. 1C). At either 10 or 20 min after tracer injection the needle was removed, and the animals were positioned supine for perfusion and fixation.

The animals were perfused by intracardiac injection of 2000 IU Heparin in 400 mL ice-cold phosphate buffered saline, followed by 500 mL of 4% paraformaldehyde (Lancaster Synthesis, Pelham, New Hampshire) in 0.1 M phosphate buffered saline (PBS) pH 7.4 at a flow rate of 50 mL/min. The spinal cord was dissected out and post-fixed in 4% paraformaldehyde in 0.1 M PBS overnight.

### Ex vivo macroscopic imaging of tracer

After post fixation, white-light and fluorescence images of the spinal cord were captured using a small animal optical imaging system (MS FX PRO Bruker, Billerica, MA). The intensity of the OA-647 signal was detected by the fluorescence camera, set at an excitation wavelength of 630 nm and emission wavelength of 700 nm. White–light images were captured to facilitate easy identification of spinal cord segments (Fig. 1D). Fluorescent images of whole spinal cord and brain were captured with an exposure time of 4 s (Fig. 1D).

### Ex vivo microscopic imaging of tracer

The spinal cord segments from C3 to T3 were dissected, cryoprotected in 30% sucrose in 0.1 M PBS pH 7.4 for 48 h and embedded in OCT compound (ProSciTech Pty Ltd, QLD, Australia). Spinal cord sections were cut transversely at 10 µm on a cryostat (Leica CM 1950 Cryostat, Amtzell, Germany). Sections were thawed in a 37 °C oven for 10 min, then washed twice for 10 min in Tris phosphate buffered saline (TPBS = 0.05 M PBS + 0.01 M TRIS). The sections were then treated with 50% ethanol/TPBS for 20 min, followed by three 10 min washes in TPBS. DAPI (1 µg/mL) was applied to each slide, incubated for 1 min to visualize the cell nuclei and then washed twice for 10 min. The sections were then coverslipped with fluorescence mounting medium (DAKO, S3023, Carpinteria, CA, US). The sections were imaged with a Zeiss Axio Imager Z2 microscope (Carl Zeiss Microimaging GmbH, Germany). Images were acquired from C3 to T3 for quantitative image analysis. All images were taken at 20× magnification and exposure times were kept constant.

### Ex vivo microscopic imaging of tracer co-localized with blood vessels

In addition to the 10 µm transverse spinal cord sections collected from C3 to T3, 40 µm sections were also cryosectioned for immunostaining with blood vessel markers. Endothelial cells were identified using a mouse anti-endothelial cell monoclonal antibody (1:100; RECA-1, ab9774, Abcam, Australia); smooth muscle cells of arteries and arterioles were identified using a mouse monoclonal anti-actin, α-smooth muscle-Cy3 antibody (1:400; SMA-Cy3, C6198, Sigma-Aldrich, USA). Immunofluorescence staining proceeded as follows. Sections were thawed in a 37 °C oven for 30 min, then washed twice for 10 min in TPBS. The sections were then treated with 50% ethanol/TPBS for 20 min followed by three 10 min washes in TPBS and incubated with 15% normal donkey serum (NDS) in TPBS pH 7.4 for 60 min. Spinal cord sections were incubated with RECA-1 overnight at 4 °C. The following day, sections were left for 2 h at room temperature before two 10 min rinses with TPBS and incubated with anti-mouse IgG Alexa Fluor 488 (1:400, A–11034, Molecular Probes, Eugene, Oregon, USA) diluted in 4% NDS/TPBS for 60 min at room temperature. The sections were rinsed with two 5 min washes in TPBS and incubated with SMA-Cy3 at 37 °C for 30 min, followed by two 10 min washes and cover slipped with fluorescence mounting medium (DAKO, Carpinteria, California, USA). The primary or secondary antibody was omitted in negative controls. Fluorescence images were taken with a digital camera (Zeiss Z1, Gottingen, Germany), and processed using Zeiss Axiovision software. All images were taken at 20× magnification and exposure times were kept constant. High magnification images of blood vessels were taken using a laser scanning confocal microscope (Zeiss LSM880, Gottingen, Germany), and processed using Zeiss Zen 2012 (black edition).

### Image analysis

Images were analyzed with Image J [32]. Macroscopic images of the brain and spinal cord were analyzed by overlaying the fluorescence image and reflected white-light image to allow spinal nerve roots to be identified, then mean fluorescence intensity was measured in each spinal segment from C2 to T8. For microscopic images, mean fluorescence intensity was measured in sections taken from spinal cord segments C3–T3. At least five spinal cord sections were analyzed per spinal level and the results for each spinal level were averaged. A region of interest outside the spinal cord was used to subtract background. Measurements were then made of the whole white matter and the whole gray matter using the manual tracing and segmentation tool in Image J. The borders of the gray matter, white matter and meninges were identified using the DAPI or RECA channel (see Additional file 1). Assessment of the distribution of CSF tracer relative to blood vessels was performed on 28 animals using Zeiss Axiovision software. Three spinal cord sections were assessed for each spinal level C3, C5, C7, T1 and T3 per animal, with a minimum of three animals per experimental group (n = 3 for all groups except 6 weeks, 10 and 20 min constriction cohorts, where n = 5 for each group).

### Classification of blood vessels

Blood vessels were classified according to diameter and by the presence or absence of smooth muscle cells (as identified by α-smooth muscle actin immunohistochemistry). *Capillaries* were identified as blood vessels less than 6.5 µm in diameter that lacked smooth muscle cells. *Arteries* and *arterioles* were identified as vessels of any size with strong α–smooth muscle immunostaining. *Veins* and *venules* were identified as blood vessels larger than 6.5 µm in diameter without a complete smooth muscle cell layer.

### Statistical analysis

Mean fluorescence intensity in sections from control and constriction animals were compared using analysis of variance (ANOVA) and adjusted for multiple comparison using Bonferroni’s post hoc tests. A *p* value of less than 0.05 was considered significant. All values are expressed as mean ± standard error of the mean.

## Results

### Surgery and post-operative observations

The extradural constriction surgery commonly caused neurological deficits, including hindlimb and forelimb weakness, urinary retention, and hematuria. In the immediate week post-surgery, bladders were manually expressed and animals received analgesia daily until neurological symptoms had abated, usually 3–5 days post-surgery. Out of 28 animals that underwent constriction surgery, 1 animal died due to urinary tract infection and 2 animals were euthanized due to severe neurological deficits within the first 48 h. No complications were observed in the control animals.

### Macroscopic imaging—CSF tracer distribution along the neuraxis

**Fig. 2:**
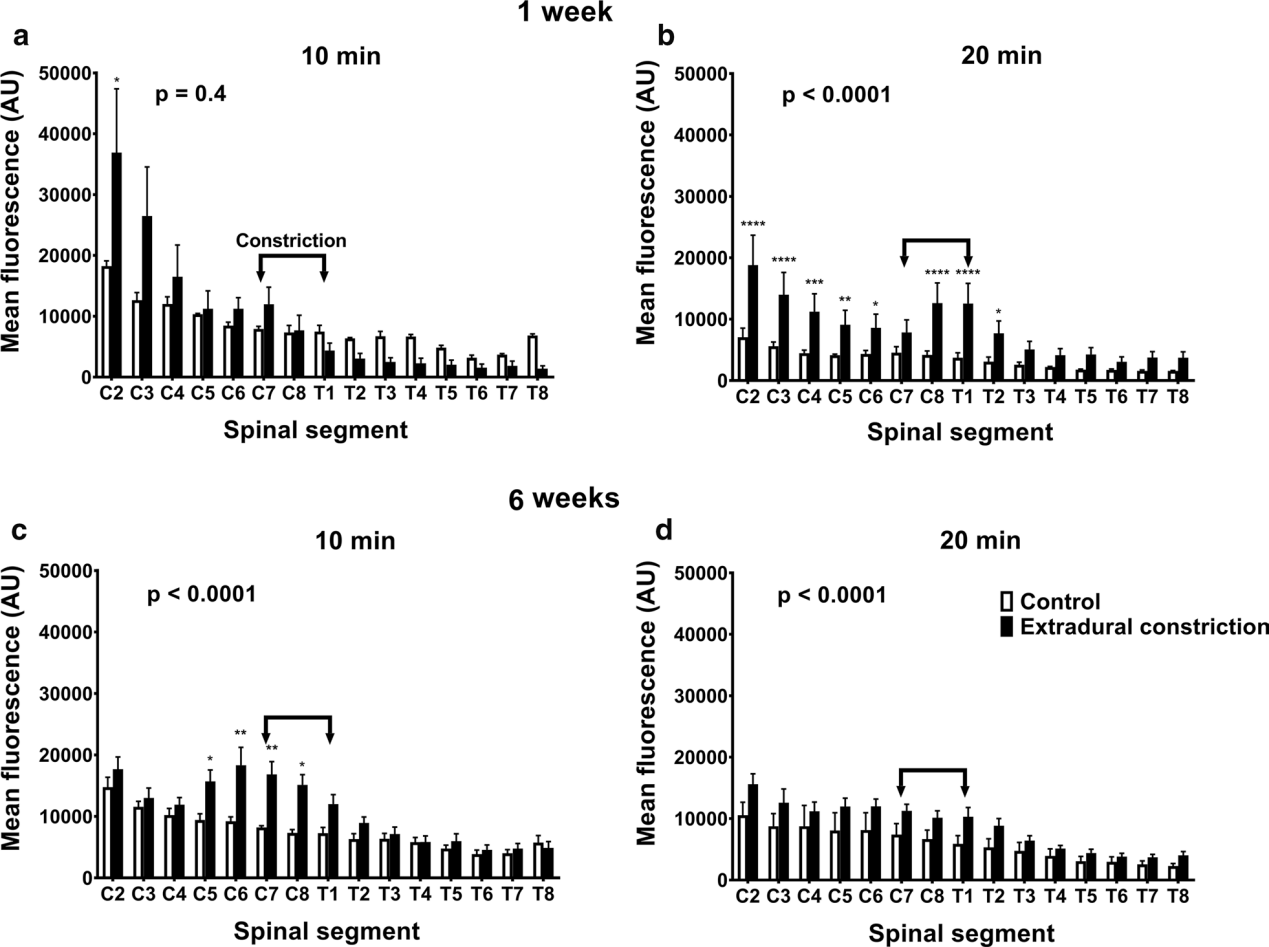
Macroscopic distribution of CSF tracer in the spinal cord 1 and 6 weeks post-constriction surgery. Graphs show mean fluorescence in each spinal segment measured macroscopically from (**a**, **b**) 1 week post-surgery in control (n = 4) and extradural constriction (n = 6 per group) animals and (**c**, **d**) 6 weeks post-surgery in control (n = 3 per group) and extradural constriction (10 min: n = 5; 20 min: n = 6) animals. Results are shown as mean fluorescence ± SEM, given as arbitrary units (AU). **a**, **c** 10 min after cisterna magna injection of CSF tracer, OA-647 (two-way ANOVA, [**a**] p = 0.4, [**c**] p < 0.0001; Bonferroni’s multiple comparison test, *p < 0.05, **p < 0.01). **b**, **d** 20 min after cisterna magna injection of CSF tracer (two-way ANOVA, p < 0.0001 for both; Bonferroni’s multiple comparison test, ****p < 0.0001, ***p < 0.001, **p < 0.01, *p < 0.05)

Detectable differences in the distribution and fluorescence intensity of CSF tracer were observed in spinal cords of animals from different treatment groups, at both 1 and 6 weeks after the initial surgery (Fig. 2).

Generally, in control animals 10 min after tracer injection, the highest intensity of CSF tracer was observed in the brain and intensity decreased gradually in the caudal direction. At 20 min, a similar pattern was observed, but in general the signal intensity was uniformly lower. In animals with a subarachnoid space obstruction the intensity of tracer fluorescence was notably increased throughout the neuraxis compared to control animals, but with a similar distribution pattern (see Additional file 2).

### 1 week post-surgery

One week after the extradural constriction surgery, and 10 min after tracer injection, rostro–caudal spread of CSF tracer was not significantly different to the control. However, post hoc comparison at the most rostral spinal segment analyzed, C2, showed significantly higher fluorescence intensity in animals with an extradural constriction (p < 0.05, Fig. 2a). Twenty minutes after tracer injection, a significant increase in rostro-caudal CSF tracer spread was reported in extradural constriction animals compared to controls (p < 0.0001, Fig. 2b; see Additional file 2). Here, CSF tracer fluorescence intensity in spinal segments C2–C6 and C8–T2 was significantly higher in constriction animals (p < 0.0001, p < 0.001, p < 0.01, p < 0.05).

### 6 weeks post-surgery

At 6 weeks post-surgery, the fluorescence intensity was significantly higher in animals with extradural constriction compared to control animals, both at 10 min (p < 0.0001) and 20 min (p < 0.0001) post–tracer injection (Fig. 2c, d; see Additional file 2). In most spinal segments, higher fluorescence intensity was reported in extradural constriction animals compared to controls, and this was significant at 10 min post-injection in spinal segments C5–C8 (p < 0.01, p < 0.05, Fig. 2c).

### Microscopic imaging—CSF tracer distribution within the spinal cord

**Fig. 3:**
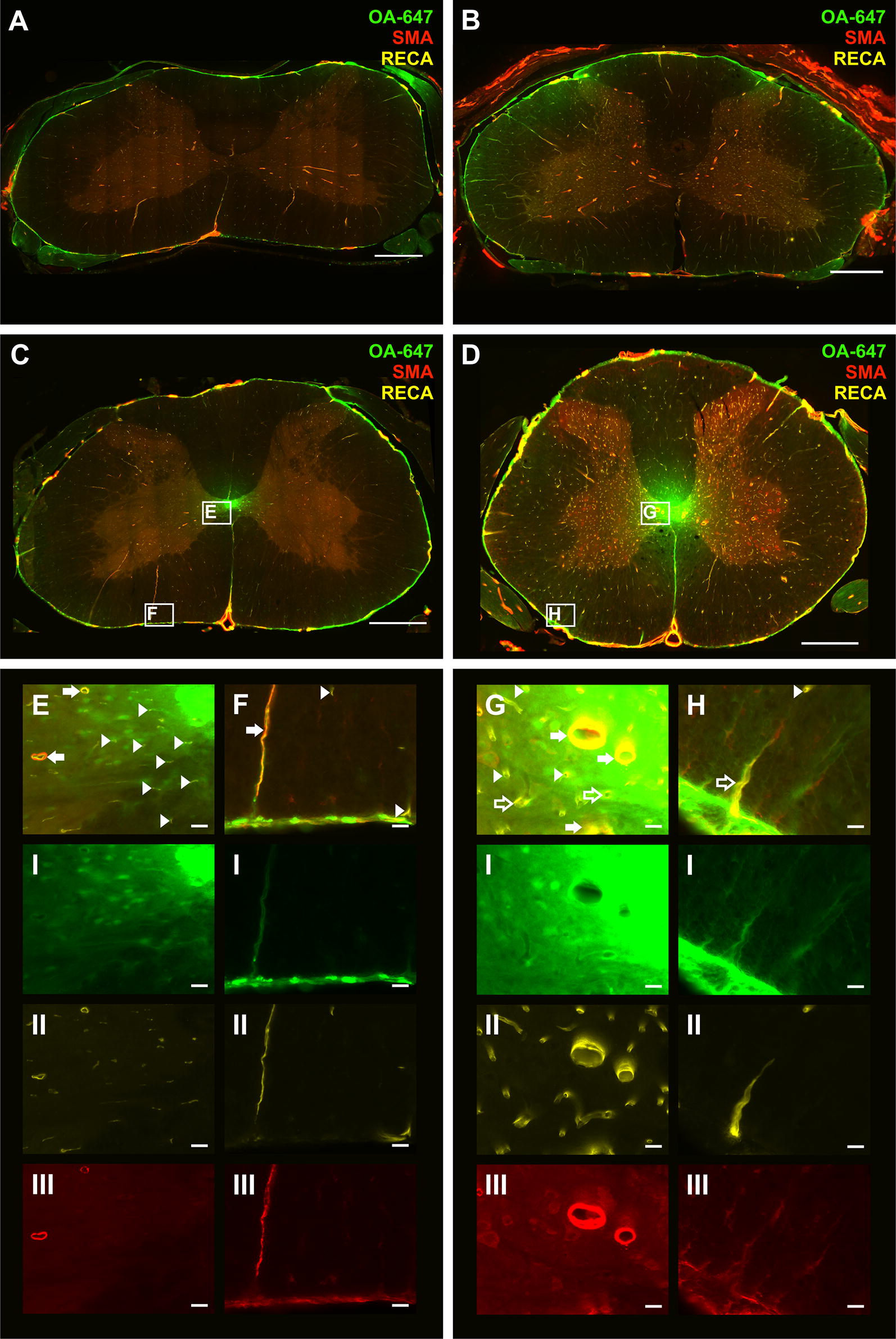
CSF tracer distribution around blood vessels in control and constriction animals at 10 min post-injection. Representative micrographs from control (**A**, **C**) and constriction (**B**, **D**) animals at 1 week (**A**, **B**) or 6 weeks (**C**, **D**) post-surgery, sacrificed 10 min after injection of tracer into the cisterna magna. Insets show tracer (OA-647) distribution in the central gray matter (**E**, **G**) and peripheral white matter (**F**, **H**) and co-localized to arterioles (arrows), capillaries (arrowheads) and venules (open arrows). Individual channels from insets **E**–**H** are also shown: OA-647 tracer (I), rat endothelial cell antigen (RECA, II) and smooth muscle actin (SMA, III). Scale bars are 500 µm (**A**–**D**) and 20 µm (**E**–**H**)

**Fig. 4:**
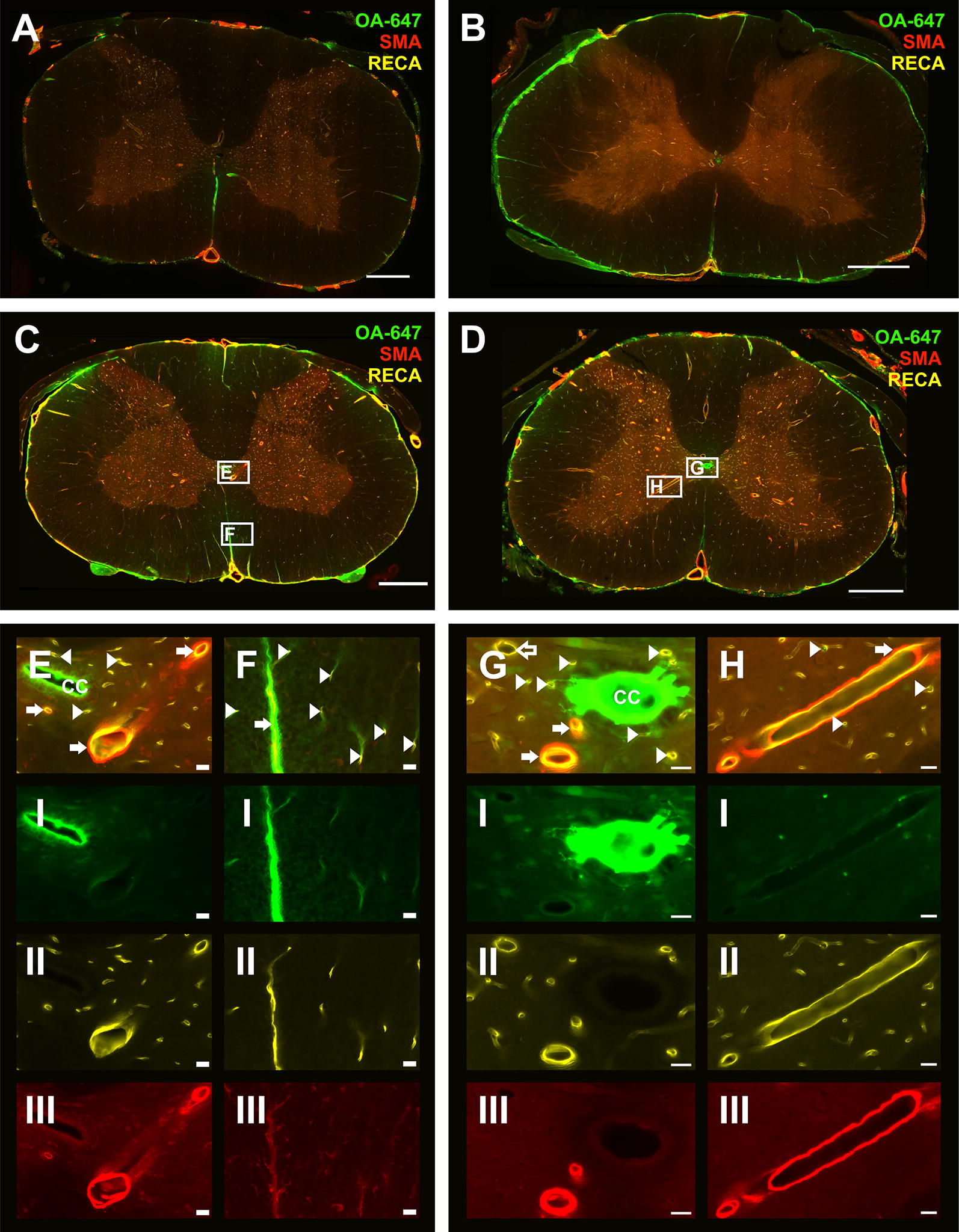
CSF tracer distribution around blood vessels in control and constriction animals at 20 min post-injection. Representative micrographs from control (**A**, ** C**) and constriction (**B**,** D**) animals at 1 week (**A**, **B**) or 6 weeks (**C**, **D**) post-surgery, sacrificed 20 min after injection of tracer into the cisterna magna. Insets show tracer (OA-647) distribution in the central canal (CC) and central gray matter (**E**, **G**, **H**), anterior median fissure (**F**) and co–localized to arterioles (arrows), capillaries (arrowheads) and venules (open arrow). Individual channels from insets (**E**–**H**) are also shown: OA-647 tracer (I), rat endothelial cell antigen (RECA, II) and smooth muscle actin (SMA, III). Scale bars are 500 µm (**A**–**D**) and 20 µm (**E**–**H**)

Within 1 and 6 weeks post-surgery groups, control and constriction cohorts showed varied tracer distribution in the spinal cord. In general, CSF tracer was predominantly concentrated around the periphery of the spinal cord, in the anterior median fissure, the posterior median sulcus, in perivascular spaces, and in the central canal at both 10 and 20 min post-injection in control animals (Figs. 3A, C and 4A, C). In animals with an extradural constriction, the tracer fluorescence intensity in the peripheral white matter of the spinal cord was generally more marked, whereas in the gray matter and central canal, the distribution of CSF tracer was similar to the corresponding controls (Figs. 3B, D and 4B, D). In both control and constriction animals, tracer was observed in a diffuse pattern in the parenchyma. This was most evident surrounding the central canal (Fig. 3E, G) Accumulation of cells and focal edema was present in several animals with extradural constriction, but a well-defined, discrete syrinx was not found (see Additional file 3).

### 1 week post-surgery

**Fig. 5:**
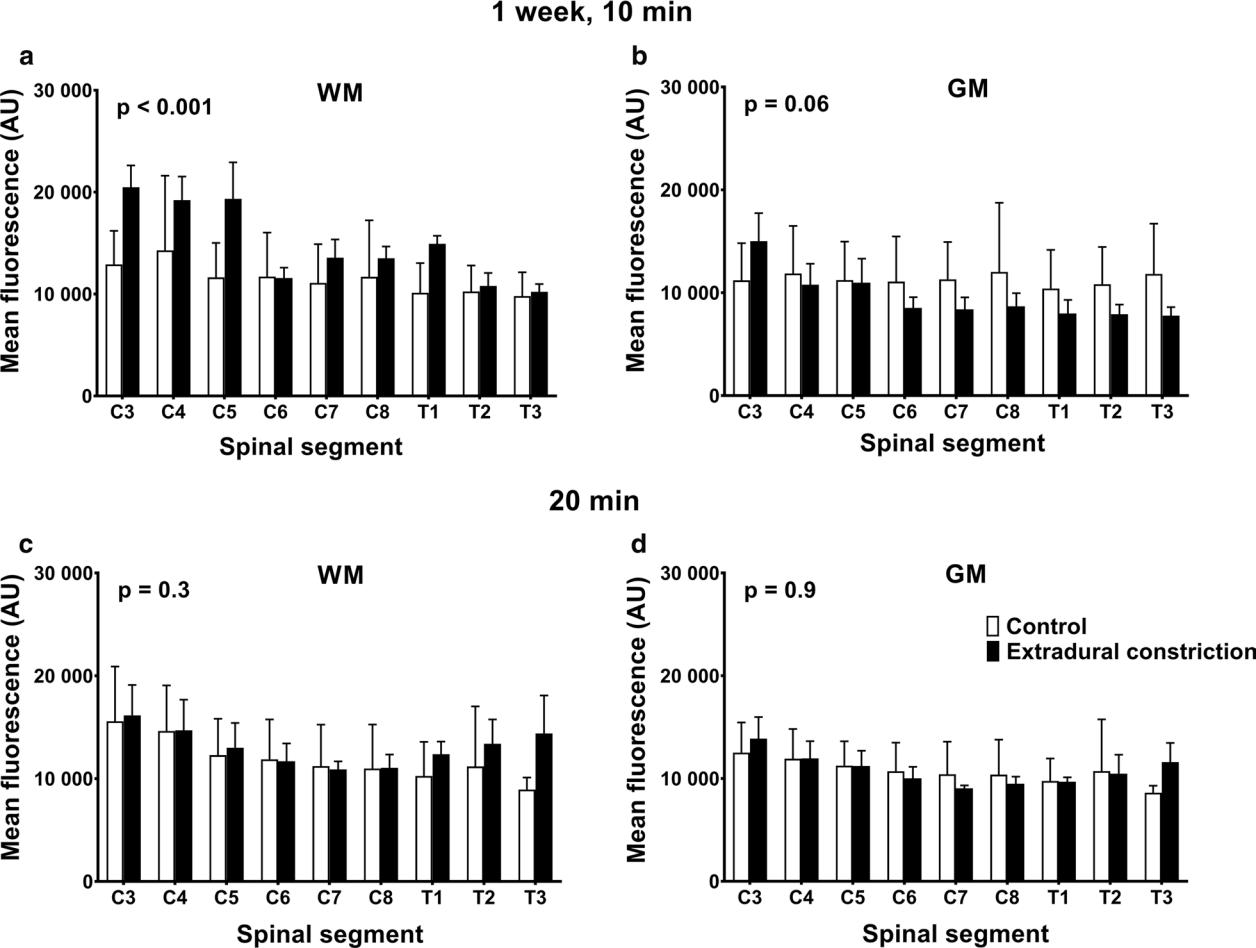
Microscopic distribution of CSF tracer in spinal cord white and gray matter 1 week post-surgery. Graphs illustrate mean fluorescence intensity measured microscopically in each spinal segment in the white matter (**a**, **c**) and gray matter (**b**, **d**) from control (n = 4 per group) and extradural constriction (n = 6 per group) animals, 10 min (**a**, **b**) and 20 min (**c**, **d**) after cisterna magna injection of CSF tracer (OA-647). Results are shown as mean fluorescence ± SEM, given as arbitrary units (AU). Statistical significance was determined using two-way ANOVA, with Bonferroni’s multiple comparison test. No statistical differences were observed in post hoc analysis. WM: white matter; GM: gray matter

Fluorescence intensity in spinal cord white matter at 1 week, 10 min after tracer injection, was significantly higher (p < 0.001) in animals with an extradural constriction (Fig. 5a). The fluorescent intensity was higher in rostral segments, although this difference did not reach statistical significance. In the gray matter, there were no significant differences in tracer intensity between control and extradural constriction animals (Fig. 5b). No significant changes were observed in the white and gray matter 20 min post-tracer injection between control and constriction animals (Fig. 5c, d).

In controls, CSF tracer was distributed primarily around capillaries and, to a lesser extent, arterioles at 10 min post-injection. By 20 min, CSF tracer was distributed around a greater number of blood vessels in the lower cervical and thoracic spinal cord. Tracer was also distributed around a small number of venules.

In constriction animals 10 min post-injection, CSF tracer appeared to be present around more blood vessels at or above the site of CSF obstruction compared to the corresponding controls. By 20 min, tracer was observed mainly in the peripheral white matter around capillaries and arterioles, as well as around a small number of venules.

### 6 weeks post-surgery

**Fig. 6:**
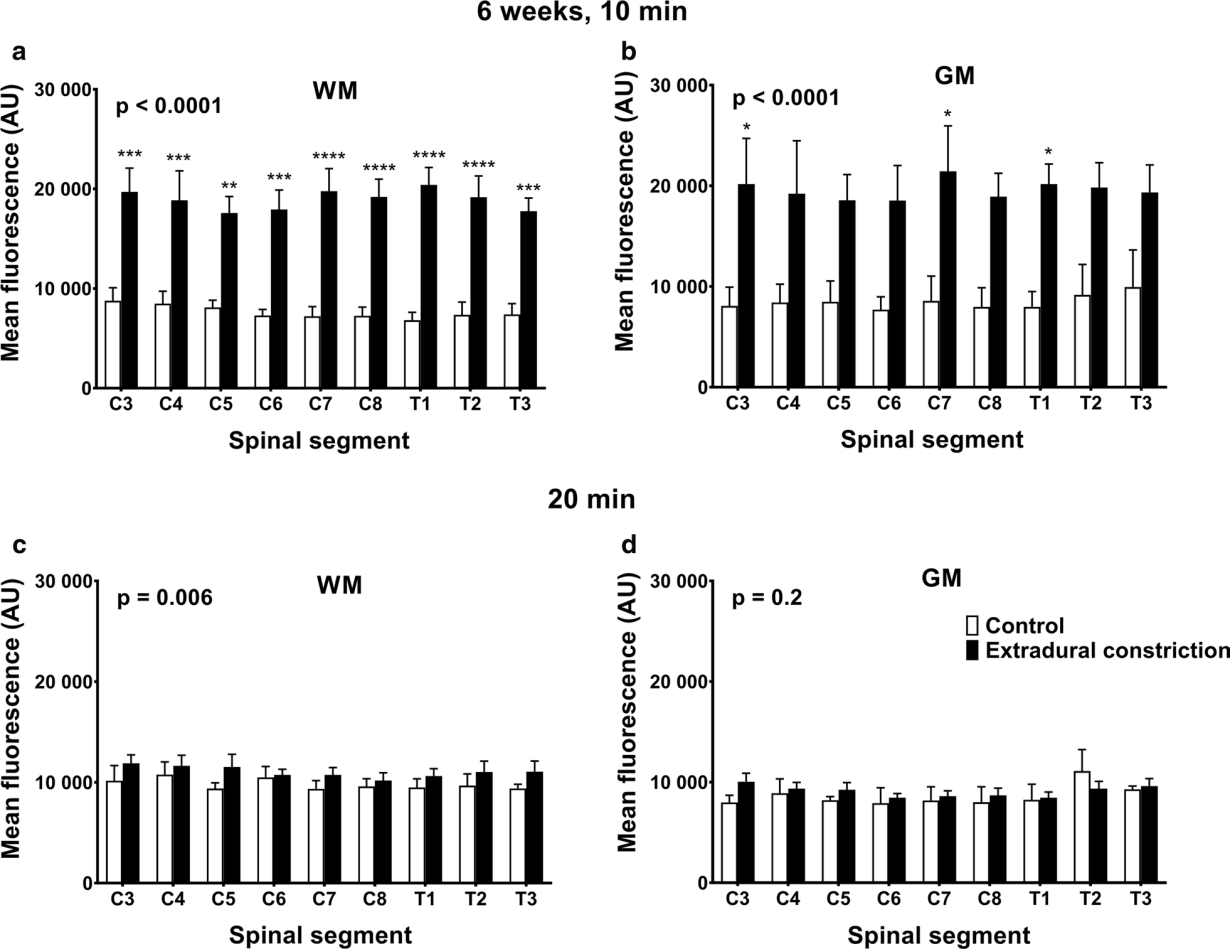
Microscopic distribution of CSF tracer in spinal cord white and gray matter 6 weeks post-surgery. Graphs illustrate mean fluorescence intensity measured microscopically in each spinal segment in the white matter (**a**, **c**) and gray matter (**b**, **d**) from control (n = 3 per group) and extradural constriction (10 min: n = 5; 20 min: n = 6) animals, 10 min (**a**, **b**) and 20 min (**c**, **d**) after cisterna magna injection of CSF tracer (OA-647). Results are shown as mean fluorescence ± SEM, given as arbitrary units (AU). Statistical significance was determined using two-way ANOVA, with Bonferroni’s multiple comparison test ****p < 0.0001, ***p < 0.001, **p < 0.01, *p < 0.05. WM: white matter; GM: gray matter

At 10 min post-injection, rostro-caudal fluorescence intensity was significantly higher in constriction animals compared to controls in white and gray matter (p < 0.0001), and post hoc analysis reached significance in the white matter of all spinal segments and in the gray matter of spinal segments C3, C7 and T1 (Fig. 6a, b). At 20 min, rostro-caudal fluorescence intensity was significantly higher in the white matter in constriction animals compared to controls (p = 0.006), although this difference was less pronounced (Fig. 6c). There was no significant difference in the gray matter at 20 min (Fig. 6d).

In control animals at 10 min post-injection, CSF tracer was mainly distributed around arterioles and capillaries (Fig. 3E, F). At 20 min post-injection, tracer was localized similarly around arterioles and capillaries (Fig. 4E, F). CSF tracer was also distributed around a small number of venules in control animals at both 10 and 20 min post-injection.

**Fig. 7:**
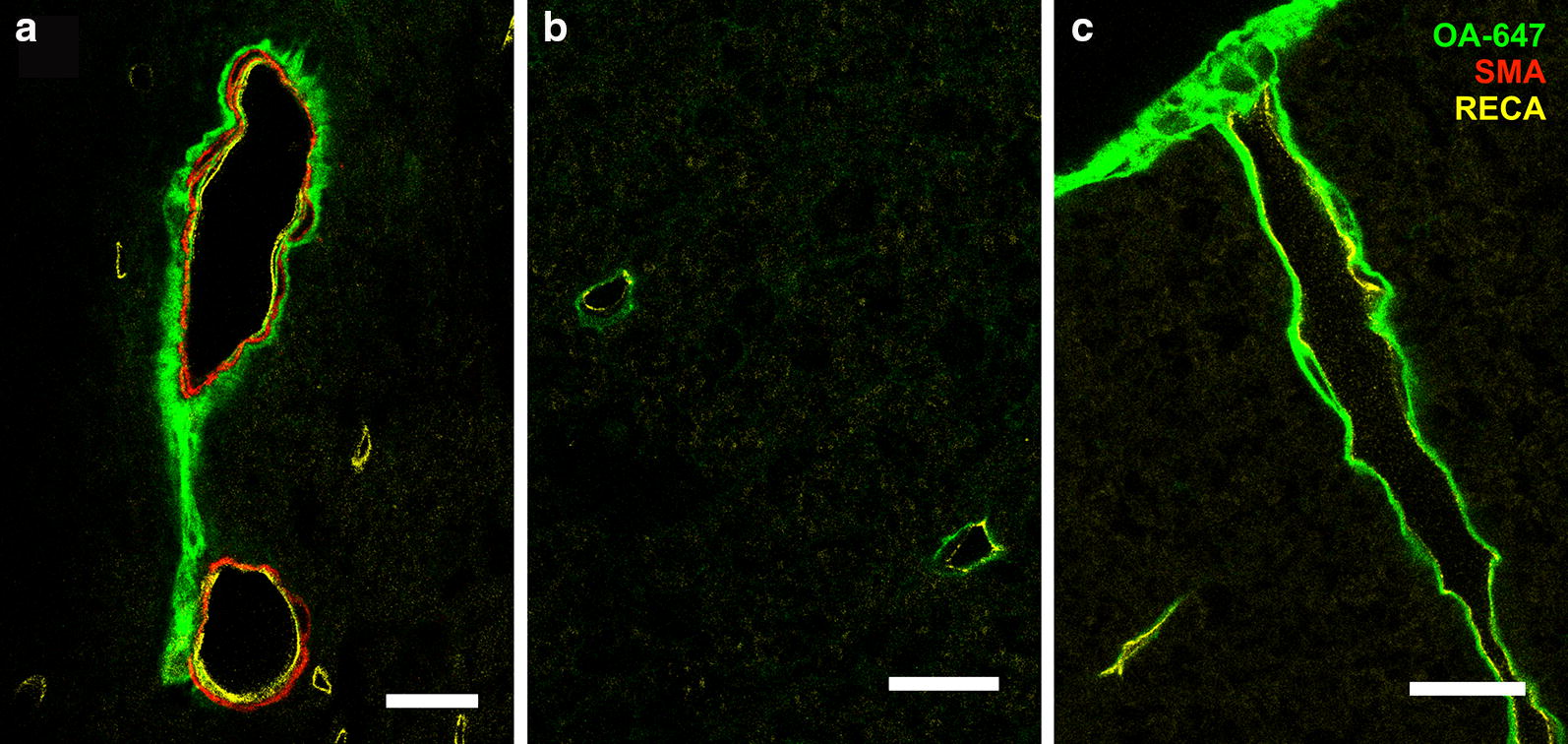
CSF tracer distribution around blood vessels at 6 weeks post-surgery. Confocal micrographs of blood vessels in transverse sections taken from animals at 6 weeks post-surgery, sacrificed 20 min after injection of tracer (OA-647) into the cisterna magna. Tracer is localized to **a** the perivascular space and smooth muscle cell basement membrane of an arteriole located in the central gray matter, **b** what is presumably the basement membrane of capillaries and **c** the perivascular space of a venule located in the peripheral white matter. Images are merged micrographs of OA-647 tracer, smooth muscle actin (SMA) and rat endothelial cell antigen (RECA) channels. Scale bars are 20 µm

In constriction animals 10 min after injection (Fig. 3D), more capillaries, as well as arterioles and venules, were associated with tracer compared to the control group (Fig. 3C). This was particularly evident at or just above the site of constriction. At 20 min post-injection (Fig. 4D) the tracer distribution around vessels was comparable to the 10 min constriction group (Fig. 3D). Tracer was predominantly observed in the perivascular spaces of venules and arterioles as well as, presumably, the basement membrane of capillaries (Fig. 7).

## Discussion

The present study investigated the effect of a subarachnoid space obstruction on fluid flow in the rat spinal cord by studying the distribution of a fluorescent tracer injected into the CSF circulation. At the macroscopic level, increased fluorescence intensity was detected at all time points, in the constriction animals compared to controls, with the increase most apparent rostral to the constriction. In contrast, microscopic analysis did not find consistently increased tracer deposition within the spinal cord parenchyma until 6 weeks after constriction, at 10 min but not 20 min post-injection. These results suggest that there is a transient rostro-caudal increase in fluid in the spinal cord white and gray matter, due to an increased inflow, a reduced outflow, or both, in the region of subarachnoid space obstruction. Importantly, co-localization of tracer with all vessel types at 10 and 20 min time points, irrespective of subarachnoid space obstruction, is in opposition to the ‘glymphatic theory’.

The macroscopic analysis of CSF tracer distribution 1 week post-surgery demonstrated a significant increase in the fluorescence intensity at 20 min post-tracer injection in animals with subarachnoid space constriction, however in the microscopic analysis, there was no significant difference in tracer deposition at this timepoint. This could be because the macroscopic imaging method is detecting changes in meningeal tracer deposition, whereas the microscopic analysis of tissue sections excluded anything external to the spinal cord parenchyma, such as the meninges and the subarachnoid space.

Differences in the distribution of CSF tracer between 1 and 6 weeks post–surgery were also observed. The severe neurological deficits observed in animals up to 5 days post-constriction surgery suggest that the initial constriction surgery caused swelling and inflammation of the spinal cord. At 1 week after constriction surgery, these changes may not have completely subsided, whereas by 6 weeks post-surgery, it is likely that the swelling and inflammation will have completely resolved. At this stage, the effect of constriction may not be as severe or complete as it was at 1 week and it is possible that the subarachnoid space is only partially obstructed.

Based on the microscopic analysis, CSF obstruction led to increased tracer fluorescence within the spinal cord 6 weeks post-surgery, most notably 10 min after injection. These results suggest that in the presence of a subarachnoid obstruction it takes longer for the tracer to clear from the spinal cord parenchyma. Although significantly higher levels of fluorescence were observed in white matter 20 min after injection, this was not as pronounced, suggesting that clearance of CSF tracer has occurred. If we assume that at 1 week there is a complete obstruction of CSF flow in the subarachnoid space and at 6 weeks there is only a partial obstruction, it is possible that the greatest impact on CSF flow into the spinal cord is due to a restriction or delay in flow rather than complete obstruction driving more CSF into the spinal cord. This is consistent with the findings of Brodbelt and colleagues [33]. The authors investigated CSF flow in an excitotoxic model of post-traumatic syringomyelia with insertion of a lumboperitoneal shunt to investigate the effect of re-establishing caudal subarachnoid space compliance as opposed to tethering or the obstructive effects of arachnoiditis. The authors found that local CSF flow into the spinal cord at the level of arachnoiditis was unchanged after shunt insertion. They suggested that localized changes in compliance, rather than total CSF obstruction from arachnoiditis, affects CSF flow into the spinal cord [33]. Indeed, in a coupled modelling study of CSF and cardiovascular systems, pressure and flow of blood and CSF were greatly impacted by the vascular anatomy of the spinal cord and the spinal subarachnoid space [34]. Martin and colleagues projected that the variable, rostro-caudal compliance in the craniospinal subarachnoid space could also account for a large amount of perivascular absorption of CSF compared with total CSF produced [34].

In 2010 Bilston and colleagues conducted a computational modelling study and established that a partial obstruction to the spinal subarachnoid space is sufficient in delaying the CSF waveform [35]. According to their theory of pressure phase lag, an increase in resistance to CSF flow in the subarachnoid space causes the normal CSF pressure waveform to lag, which results in a higher CSF pressure in the subarachnoid space for 25% of the cardiac cycle after systole. It has been suggested that perivascular spaces of arterioles at diastole of the cardiac cycle are at their widest and in systole they are at their narrowest, so these spaces are likely increasing in width after the systolic pulse wave has passed through. The delayed CSF pulse wave coupled with an unaffected arterial pulse wave could potentially drive more fluid along widening perivascular spaces and into the spinal cord [35]. This is also consistent with the modelling of posterior (partial) arachnoiditis reported by Cheng and colleagues [36]. The authors concluded that the change in the timing of the CSF waveform with respect to the cardiac cycle caused a bidirectional flow in the subarachnoid space not seen in the circumferential arachnoiditis model [36]. Further modelling of a cervical subarachnoid space obstruction by Støverud and colleagues demonstrated a mistiming of bidirectional flow at the level of the obstruction and an overall increase in velocity and pressure gradients of CSF. Importantly, the phase lag between pressure and velocity decreased [37]. These findings add credence to the theory that changes of the relative timing of the CSF pulse wave to the arterial pulse wave may drive more fluid into the spinal cord.

The microscopic analysis of tracer distribution in the current study demonstrated that the subarachnoid constriction had a greater effect on CSF flow in the white matter, and this was most notable 10 min post tracer injection. This could be due to the fact that after the injection, the tracer is predominantly moving from the subarachnoid space into the white matter, either by transpial diffusion or via perivascular spaces. Subsequently, it flows into the gray matter, however, 10 min may not be long enough for tracer to reach the gray matter. However, tracer in the spinal subarachnoid space can also enter the central gray matter ECS via the anterior median fissure, and the penetrating perivascular spaces then funnel into the central canal or traverse towards the peripheral white matter [16, 17]. It is possible that with an obstructed subarachnoid space due to the extradural constriction, this pathway of tracer flow is disturbed. Since the effect of the subarachnoid space obstruction was greatest at the earlier post-injection time, this suggests that the extradural constriction did alter the normal flow of CSF in the subarachnoid space, leading to a temporary increase in fluid within the spinal cord parenchyma. There appears to be a delayed compensatory pathway that allows clearance of excess fluid from the spinal cord. The maintenance of fluid homeostasis in the spinal cord relies on the balance between inflow and outflow, yet perhaps in the presence of an obstruction these outflow pathways become compromised, leading to edema and subsequent syrinx formation. Previous investigations into the pathogenesis of syringomyelia proposed that a syrinx forms due to an accumulation of extracellular fluid that is unable to be removed [38]. It was suggested that the subarachnoid pressure exceeds the intramedullary pressure leading to a blockage of the perivascular spaces in the white matter. This in turn causes fluid to accumulate, producing edema and eventually syringomyelia below the level of subarachnoid scar/obstruction [38].

In the current study, syrinx cavities were not observed in animals with an extradural constriction. However, cellular accumulation accompanied by focal edema in the deep white matter and anterior horns of the gray matter was present at the level of constriction in a few animals (see Additional file 3). This indicates that in this model, a syrinx may take longer to develop than the 6 week time point investigated in this study. Josephson and colleagues indeed found fluid accumulation and syrinx formation at 8 weeks in a rat model of spinal thecal sac constriction [31]. Yet, lack of syrinx formation in the present study may suggest that syringomyelia is not simply caused by an obstruction to subarachnoid CSF flow. The pathophysiology appears far more complex. There is some evidence that molecular changes may be involved in fluid accumulation in the spinal cord [39, 40]. Nesic and colleagues suggested that increased AQP4 expression in the spinal cord following injury may lead to edema and swelling and this may contribute, at least in part, to initial cyst formation [39]. Hemley and colleagues reported a significant increase in AQP4 expression at the level of syrinx in the post-traumatic syringomyelia model and suggested that there may be a relationship between AQP4 expression levels and fluid accumulation in the spinal cord [41]. Concurrently, Najafi and colleagues demonstrated that in astrocytes adjacent to post-traumatic syrinx cavities, the expression of inwardly rectifying potassium channel 4.1 was significantly decreased [42]. Altered expression or distribution of water and ion channels could represent a contributing factor to the observed alterations in fluid flow.

In the brain, it has been reported that CSF flow into and out of parenchyma occurs via bulk flow that is dependent on the presence of AQP4 at astrocytic endfeet lining peri-arterial and peri-venular spaces [12]. Bulk flow of ISF has previously been demonstrated in the brain under physiological and hyperosmolar conditions [43]. In contrast to the ‘glymphatic’ theory proposed by Iliff and colleagues [12], other studies have indicated that tracers injected into brain parenchyma are removed from the brain via diffusion in the ECS and then via a perivascular pathway, through arterial and capillary basement membranes [13]. More recent studies have shown that bulk flow in the brain may not be required for a glymphatic system of solute transport [44]. Asgari and colleagues used computational modelling of arterial pulsations to show that the fast solute transport along periarterial spaces in the brain could occur due to fluid dispersion, rather than bulk flow [44]. This dispersion of fluids and solutes involves periarterial mixing and diffusion into brain ECS. Further modelling of the brain ECS has suggested that diffusion, under normal physiological conditions, is the main determinant for solute transport [45, 46]. Through the authors’ modelling, an advective solute transport in a glymphatic system was not supported. Indeed, an increasing body of evidence, inclusive of the present study, are in direct conflict with the glymphatic hypothesis [44–50]. These studies implicate a perivascular system of fluid transport in the brain, a pathway where perivascular spaces of macrovessels permit convective or dispersive flow and the microvessel perivascular spaces and basement membranes in conjunction with surrounding neuropil, described as the neurovascular unit, allow the regulatory CSF/ISF exchange by diffusion. Liu and colleagues recently described movement of tracer injected into the spinal cord as dependent on the diffusivity of gray and white matter [51]. Perivascular spaces around microvessels were reported as major outflow conduits [51]. Nevertheless, mechanisms of fluid flow in the spinal cord in normal and abnormal conditions remain poorly described.

In this study, co-localization of tracer with all vessel types was found in both control and constriction animals in gray and white matter at all time points. This finding suggests that fluid flow into the spinal cord does not occur preferentially along peri-arterial or peri-venular spaces. Alternatively, it may indicate perivascular mixing, representing the back and forth movement of the CSF tracer between perivascular and subarachnoid space, and may not lead to significant net transport of CSF tracer into the parenchyma itself. The lack of individual vessel type differences in perivascular tracer localization between control and constriction cohorts, irrespective of post-surgery and post-injection time points, may indicate that these pathways remain constant regardless of a disturbed flow in the subarachnoid space. However, enlargement of these spaces, which has been associated with the pathology of post-traumatic syringomyelia [52], may permit a greater volume of tracer conveyed through the perivascular network. Coupled with reduced outflow due to the constriction, an increased inflow of tracer may explain why more arterioles, capillaries, and venules were found co-localized with tracer in the 6 week constriction animals compared to the corresponding controls. This increased inflow/reduced outflow theory could also explain why white matter in constriction animals showed significantly higher fluorescence at 10 min post-injection than controls, 1 and 6 weeks post-surgery. It is also likely, given the vast number of capillaries surrounded by tracer, in both control and constriction animals, that the basement membrane is acting as either an inflow or outflow pathway, or both. If this is the case, pericapillary flow is likely to be a major pathway for fluid exchange. This has been suggested previously in studies of rodent cortex [49, 50]. This pathway of fluid exchange would suggest that capillary basement membranes act as connecting conduits between the larger, parent perivascular spaces of arterioles and venules. Indeed, a recent electron microscopic study of spinal cord perivascular spaces and their role in the transport of fluid suggested that there is a continuity between subarachnoid space and central canal of the spinal cord, via perivascular spaces, the basement membranes including capillary basement membranes, and ECS of the white matter and the central gray matter [53].

It should be noted that CSF tracer was commonly observed in the central canal of animals in all experimental groups. This is consistent with several studies demonstrating fluid flow from the spinal subarachnoid space, through the parenchyma via perivascular spaces and on to the central canal in animal models [16, 17, 51]. The central canal has been proposed as a major clearance route for extracellular fluid. Described as a sink, it is suggested to protect spinal cord parenchyma by draining neurotoxic substances [54]. It is possible that under conditions of flow obstruction within the subarachnoid space, the central canal clearance pathway may not be able to compensate for increased ISF volume. Coupled with volume changes in perivascular spaces, this unresolved ISF volume increase may precede syrinx formation.

The study of tracer distribution from subarachnoid space to spinal cord parenchyma is a valid and effective method for assessing CSF flow patterns. However, assumptions are made about fluid pathways, based on deposition profiles, representing a snapshot in time. The extradural constriction model used in this study is a valuable model of subarachnoid space obstruction. However, the ischemia, caused by the constriction of the vein and surrounding vessels by the suture, may have an unmeasured effect of disrupting fluid movement and changing flow pathways. Disruption to cervical lymphatic drainage at spinal nerve root sheaths may also result from the extradural constriction. Finally, as maximum sample size across any experimental group was 6 animals with a minimum of 3, increasing experimental group cohorts in future studies may elicit more robust results.

## Conclusions

This CSF tracer study demonstrated that a subarachnoid space obstruction significantly increases CSF tracer fluorescence intensity in rat spinal cord tissue. This provides evidence that a restriction of, or disruption to, CSF flow leads to an increased influx of fluid in the spinal cord, with possibly a concomitant disruption to efflux pathways. The vast network of perivascular spaces of arterioles, venules and the connecting capillary basement membranes are likely contributors to this exchange pathway in both normal physiological conditions and when there is abnormal CSF circulation. The findings of this study suggest that even without a complete obstruction, an imbalance between inflow and outflow volumes (of possible perivascular origin) may precede fluid accumulation. Further investigations are needed to clarify the mechanisms and physiological factors that result in fluid accumulation pathologies such as post-traumatic syringomyelia.

## Additional files

**Additional file 1.** Manual segmentation and measurement of spinal cord gray and white matter. Transverse micrographs of spinal cord illustrate manual tracing and segmentation of gray and white matter for measurement in ImageJ software. The transverse spinal cord was manually traced to exclude meningeal fluorescence, and gray matter was outlined as a separate region of interest (A). White matter fluorescence was calculated by measuring total spinal cord fluorescence and subtracting the gray matter fluorescence (B). Gray matter fluorescence was isolated as a region of interest and measured (C). Scale bars are 500 µm.

**Additional file 2.** Macroscopic fluorescence images of brain and spinal cord from 1 and 6 week cohorts. Macroscopic images of dissected central nervous system (CNS) illustrate representative fluorescence intensities from individual study groups. Whole CNS fluorescence images are captured at 1 or 6 weeks after extradural constriction surgery (constrict.) or laminectomy–only (control) and 10 or 20 min after intracisternal injection of a CSF tracer (OA–647). In general, fluorescence in spinal cords of constriction animals appears more intense than corresponding control animals.

**Additional file 3.** Cellular accumulation and focal edema identified in extradural constriction animals 1 and 6 weeks post-surgery. Representative micrographs at the level of the extradural constriction (C7–T1) from animals 1 and 6 weeks post-surgery after injection of CSF tracer (OA-647). Low magnification micrographs demonstrate focal edema and the infiltration/accumulation of cells within the deep anterior white matter (A–C) and anterior horns of the gray matter (D, E). High magnification insets (a–e) illustrate hyperintensity of smooth muscle actin (SMA) and rat endothelial cell antigen (RECA) staining, especially evident in the anterior horns (d, e). Scale bars are 500 µm (A–E) and 50 µm (a–e).

## Abbreviations

ANOVA: analysis of variance
AQP4: aquaporin-4
CNS: central nervous system
CSF: cerebrospinal fluid
ECS: extracellular space
HRP: horseradish peroxidase
ISF: interstitial fluid
MRI: magnetic resonance imaging
PBS: phosphate buffered saline
RECA: rat endothelial cell antigen
SEM: standard error of the mean
SMA: smooth muscle actin
TPBS: tris-phosphate buffered saline
