# Supplementary figures and images for: Effect of extradural constriction on CSF flow in rat spinal cord
Source: Fluids Barriers CNS. 2019 Mar 26;16:7. doi: 10.1186/s12987-019-0127-8 (PMC6434898; doi:10.1186/s12987-019-0127-8)

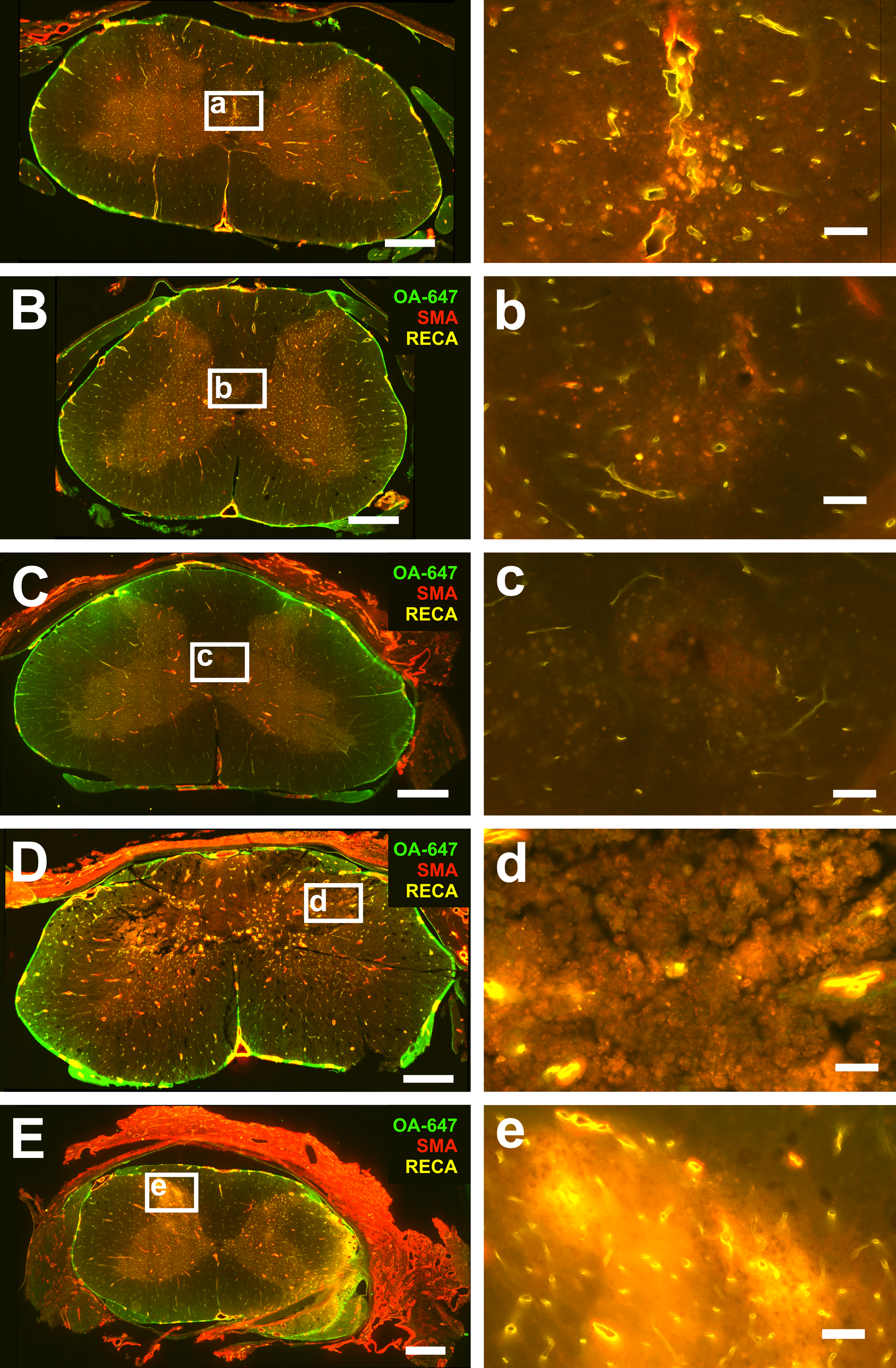

Supplement: Supplementary file 3 — Additional file 3. Cellular accumulation and focal edema identified in extradural constriction animals 1 and 6 weeks post-surgery. Representative micrographs at the level of the extradural constriction (C7–T1) from animals 1 and 6 weeks post-surgery after injection of CSF tracer (OA-647). Low magnification micrographs demonstrate focal edema and the infiltration/accumulation of cells within the deep anterior white matter (A–C) and anterior horns of the gray matter (D, E). High magnification insets (a–e) illustrate hyperintensity of smooth muscle actin (SMA) and rat endothelial cell antigen (RECA) staining, especially evident in the anterior horns (d, e). Scale bars are 500 µm (A–E) and 50 µm (a–e). [file 12987_2019_127_MOESM3_ESM.tif]
